# Endogenous carbon monoxide production in extracorporeal membrane oxygenation‐related hemolysis: potential use of point‐of‐care CO‐oximetry carboxyhemoglobin to detect hemolysis

**DOI:** 10.1002/ccr3.1351

**Published:** 2018-01-03

**Authors:** Chan Kai Man, Lam Koon Ngai

**Affiliations:** ^1^ Department of Anaesthesia and Intensive Care Prince of Wales Hospital Chinese University of Hong Kong Shatin Hong Kong; ^2^ Department of Intensive Care North District Hospital Chinese University of Hong Kong Sheung Shui Hong Kong

**Keywords:** Carbon monoxide, carboxyhemoglobin, extracorporeal membrane oxygenation, hemolysis

## Abstract

Extracorporeal membrane oxygenation (ECMO)‐related hemolysis is a rare but devastating condition. Death is inevitable without early recognition and prompt management. Endogenous carbon monoxide production, as an epiphenomenon of hemolysis, potentially allows rapid detection of such condition by use of point‐of‐care CO‐oximetry carboxyhemoglobin.

## Introduction

Hemolysis is a rapidly progressive and devastating complication of extracorporeal membrane oxygenation (ECMO). Early diagnosis is essential for aggressive management. However, plasma‐free hemoglobin (pfHb) measurement, which is the gold standard diagnostic test, requires turn over time and may not be widely available in every laboratory. Point‐of‐care test in this setting may aid rapid bedside diagnosis.

Endogenous carbon monoxide production in patients with severe hemolysis while receiving ECMO has been reported 1, 2. Carboxyhemoglobinemia in mild‐to‐moderate hemolysis related to ECMO has never been reported. We report two patients who developed moderate and severe hemolysis while receiving ECMO. A proportional endogenous carbon monoxide production in relation to the pfHb level was found in them. While carboxyhemoglobin (CO‐Hb) may worsen tissue oxygen delivery in these very sick patients, CO‐Hb detection by use of bedside point‐of‐care co‐oximetry may provide opportunity for early rapid diagnosis of ECMO‐related hemolysis.

## Case 1

A 54‐year‐old man, who was a construction site worker, was admitted for fever, malaise, myalgia, and associated diarrhea and vomiting. He had underlying poorly controlled diabetes despite gliclazide, metformin, and insulin. He developed severe acute respiratory distress syndrome and septic shock requiring mechanical ventilation and noradrenaline infusion. His condition continued to deteriorate despite FIO_2_ 1.0, PEEP 8 cmH_2_O, broad‐spectrum antibacterials, voriconazole, and 3 days of prone ventilation. Subsequent bronchoscopic examination revealed multiple ulcerative lesions in main bronchus and bronchioles. *Aspergillus fumigatus* and *Stenotrophomonas maltophilia* were grown from bronchial alveolar lavage fluid. He was managed as invasive aspergillosis and ventilator‐associated pneumonia. Liposomal amphotericin B and septrin were added. Venovenous ECMO (VV ECMO) was employed after 5 days of ICU care with PaO_2_ to FIO_2_ ratio of 60, PaCO_2_ 8.35 kPa, pH 7.217, and high‐dose noradrenaline infusion at the time of ECMO initiation.

The ECMO circuit consisted of Maquet Cardiohelp HLS system, drainage from intrahepatic vena cava via 21 French (Fr) multistage cannula in left femoral vein, a second drainage from superior vena cava (SVC) via 17‐Fr cannula in right internal jugular vein, and return to right atrium via 19‐Fr cannula in right femoral vein. A blood flow of 4.5 L/min was achieved. Heparin was not started until 12 h after ECMO due to elevated activated partial thromboplastin time (>120 sec) as a result of disseminated intravascular coagulation. Urea and Creatinine at that time were 18.3 mmol/L and 130 *μ*mol/L, respectively. CO‐Hb level was 0.3% at that time, and values were all <1.3% beforehand. Methemoglobin level was 0.4%.

On 7th day of ICU stay, hematuria was noted, shortly followed by anuria. Hemoglobin dropped from 10.0 to 8.5 g/dL. Platelet count dropped from normal to 70 × 10^9^/L. Blood biochemistries sent including indirect bilirubin, lactate dehydrogenase, and haptoglobin were all rejected by laboratory due to severely hemolyzed specimen. Serial pfHb measured 1600 mg/dL followed by unmeasurably high value (>2000 mg/dL). G6PD level was normal. There were no other drugs suspicious to cause hemolysis. A concomitant rise in CO‐Hb from 0.8% to 4.5%, and a peak of 9.1% was observed at the same time. No thrombus was seen over the oxygenator and tubings. The transmembrane pressure (TMP) of oxygenator never exceeded 30 mmHg.

Continuous renal replacement therapy (CRRT) was started, which yielded red effluent. Despite aggressive management, patient remained to be in severely shock state and succumbed after 8 days of ICU care.

## Case 2

A 65‐year‐old man, previously enjoying good past health, admitted for anterior ST‐elevated myocardial infarction with cardiogenic shock, was urgently escorted to cardiac catheterization laboratory for emergency percutaneous coronary intervention (PCI) and intra‐aortic balloon pump insertion. He developed PEA arrest while having PCI and venoarterial ECMO (VA ECMO) was employed in addition to standard cardiopulmonary resuscitation. The ECMO circuit consists of Maquet Cardiohelp HLS system, drainage from left femoral vein via 21‐Fr cannula, and return to left femoral artery via 19‐Fr cannula. PCI was considered unsuccessful, and patient received beating heart coronary artery bypass grafting, left ventricular venting, and 8‐Fr distal perfusion catheter for left lower limb perfusion. His condition was complicated by access insufficiency with venous cannula chattering requiring repeated fluid boluses, and acute renal failure requiring CRRT during the first 3 days in ICU. In addition, heparinization was subtherapeutic on 3rd day of ICU stay, and small amount of fibrin was seen on vent tubing. pfHb level was 3.2 mg/dL on the day, and CO‐Hb values were all <2% during the first 3 days. However, an increase in pfHb of 79.3 and 100.7 mg/dL was seen on 4th day and 5th day, respectively. There was a corresponding increase in CO‐Hb of 3.3% and 3.8%. Slightly pinkish effluent hemofiltrate was also observed. No thrombus was seen within the oxygenator and TMP never exceeded 33 mmHg. pfHb dropped to 4.9 mg/dL next day after intensive titration of heparin, along with a concomitant drop of CO‐Hb to 2.6%, then 2.0%. Clear CRRT effluent was observed again. CO‐Hb levels never exceeded 2.0% throughout the subsequent ICU stay. However, his condition was further complicated by upper gastrointestinal bleeding and multiorgan failure. Patient succumbed after 10 days of ICU care.

## Discussion

While carboxyhemoglobinemia is well reported as a manifestation of hemolysis in drug‐induced hemolytic anemia 3 and sickle cell anemia 4, carboxyhemoglobin is typically slightly elevated in critically ill patient, in range of 0.9–1.9% 5. We believe that the much elevated CO‐Hb levels in our patients are secondary to ECMO‐related severe hemolysis 6. With severe hemolysis, free hemoglobin is degraded by heme oxygenase (HO) to free iron, biliverdin, and carbon monoxide. Three isoforms of HO (HO‐1, HO‐2, and HO‐3) exist, and HO‐1 is inducible by heme, oxidative stress, and inhalational nitric oxide, thereby further perpetuating carbon monoxide formation. While dyshemoglobinemia in such situations further worsen oxygen delivery and increase the difficulty of pulse oximetry monitoring 7, measurement of CO‐Hb in these patients can be a useful tool for early detection of ECMO‐related hemolysis. To our knowledge, two cases of endogenous carboxyhemoglobin production related to severe hemolysis secondary to ECMO are reported 1, 2. However, there has been no report analyzing the relationship between pfHb and CO‐Hb levels.

**Figure 1:**
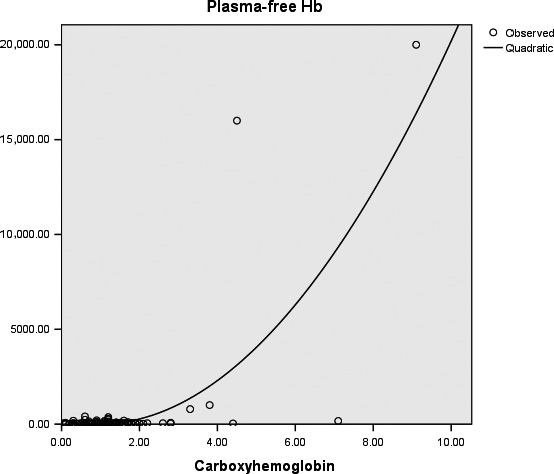
Carboxyhemoglobin (%) plot against plasma‐free hemoglobin (mg/L) in 33 consecutive patients receiving extracorporeal membrane oxygenation (ECMO) with or without clinical hemolysis.

From our result, the peak CO‐Hb value of 9.1% in patient 1, with a corresponding unmeasurably high pfHb (>2000 mg/dL), is consistent with the reported value of 9.5% and 11.6% in grossly severe hemolysis 1, 2. More importantly, the measured values of pfHb of 79.3 and 100.7 mg/dL in patient 2 and measured value of pfHb of 1600 mg/dL in patient 1 correspond linearly with their respective CO‐Hb of 3.3%, 3.8%, and 4.5% (Fig. 1). We suspect a positive correlation exists.

**Figure 2:**
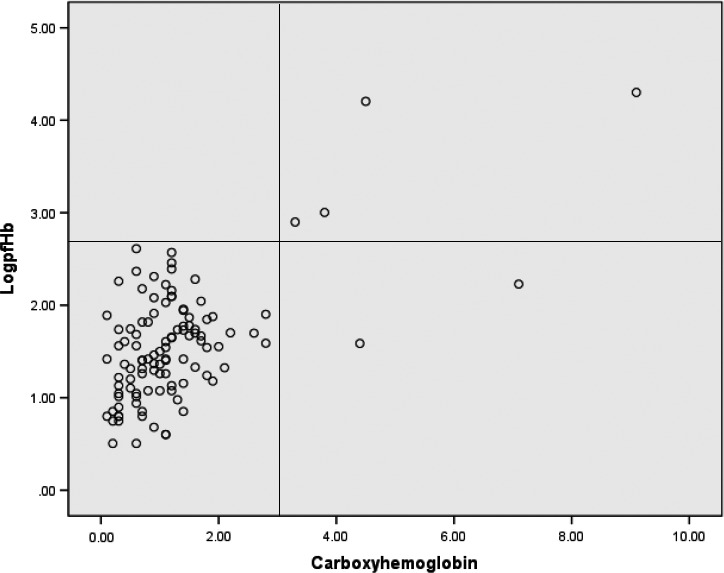
Log_10_ of plasma‐free hemoglobin (mg/L) plot against carboxyhemoglobin (%) in a cohort of 33 patients receiving extracorporeal membrane oxygenation (ECMO) with or without hemolysis. Logarithmic scale is used to better display the large range (0–20,000 mg/L) of plasma‐free hemoglobin values on a numeric scale of 0–5; the horizontal line corresponds to log_10_ value of pfHb of 500 mg/L (50 mg/dL, with reference to extracorporeal life support organization (ELSO) recommendation); the vertical line corresponds to 3% CO‐Hb. No patient with hemolysis, defined as pfHb greater than 500 mg/L, has CO‐Hb value of <3% (i.e., left upper quadrant). All, but two of the measurements of CO‐Hb values, are <3% if hemolysis is absent. The two exceptional measurements of CO‐Hb (4.4% and 7.1%) in right lower quadrant are obtained from a single patient without hemolysis.

We analyzed the pfHb and their corresponding CO‐Hb in 33 consecutive patients with or without hemolysis who received ECMO therapy in our ICU from October 2009 to February 2015, inclusive of the above two reported patients. Baseline demographic data are shown (Table 1). Hemolysis is defined as pfHb value of greater than 50 mg/dL with reference to the Extracorporeal Life Support Organization (ELSO) recommendations 8. A total of 110 pairs of measurements were recorded (Fig. 2). All, but two of the measurements of CO‐Hb values, are <3% if hemolysis is absent. The two exceptional measurements are obtained from a single patient with CO‐Hb values of 4.4% and 7.1%. All CO‐Hb values are >3% if hemolysis is present. A 3% CO‐Hb cutoff appeared to be discriminative of hemolysis (Table 1, Fisher's exact test, *P* = 0.006). A more detailed multivariate analysis would be warranted to exclude the effects of other confounding variables.

**Table 1:** Demographics data of 33 consecutive patients receiving ECMO with or without hemolysis in a single tertiary ICU from October 2009 to February 2015

Variables	Mean (Median), Percentage % (number)	Range, (IQR)
Age	44 (48)	13–77 (29–57)
Gender		
Male	42.4% (14)	
Female	57.6% (19)	
Indications of ECMO		
Respiratory causes	42.4% (14)	
Viral pneumonia	18.2% (6)	
Bacterial pneumonia	6.1% (2)	
Massive hemoptysis	6.1% (2)	
Other respiratory diagnosis	3.0% (1)	
Extrapulmonary ARDS	3.0% (1)	
Chest injuries	3.0% (1)	
Asthma	3.0% (1)	
Cardiovascular causes	57.6% (19)	
Acute myocardial infarction	6.1% (2)	
Myocarditis	9.1% (3)	
Severe valvular regurgitation/stenosis	9.1% (3)	
Postcardiotomy cardiogenic shock	27.3% (9)	
Amniotic fluid embolism	3.0% (1)	
Massive pulmonary embolism	3.0% (1)	
Severe pulmonary hypertension	3.0% (1)	
CRRT while on ECMO	50% (14)^a^	
Mode		
VV	33.3% (11)	
VA	63.6% (21)	
VV converted to VA	3.0% (1)	
Clinical signs of hemolysis while on ECMO	6.1% (2)	
Bleeding complications (overall)	24.2% (8)	
Cannulation site	3.0% (1)	
Tracheostomy site	3.0% (1)	
Sternotomy site requiring resternotomy	6.1% (2)	
Gastrointestinal	3.0% (1)	
Hemothorax	9.1% (3)	
Number of days on ECMO	8.8 (4.5)	1–37 (2–11)
Wean from ECMO	53.1% (17)^b^	
ICU length of stay (days)	14.0 (11.5)	1–50 (3–16)
Death within ICU	48.5% (16)	
Hospital death^c^	48.5% (16)	
Hemolysis^d^	6.1% (2)	
CO‐Hb <3%	0% (0)	
CO‐Hb >3%	100% (2)	
Without Hemolysis^e^	93.9% (31)	
CO‐Hb <3%	96.8% (30)	
CO‐Hb >3%	3.2% (1)	

IQR, interquartile range; ECMO, extracorporeal membrane oxygenation; ARDS, acute respiratory distress syndrome

a CRRT after ECMO; CRRT is provided after ECMO is initiated, five patients’ data are missing.

b Wean from ECMO; liberation from ECMO without death in the next 7 days and second run ECMO, one patient's data are missing.

c Hospital death; death before hospital discharge to home or a convalescent hospital.

d Hemolysis; plasma‐free Hb >50 mg/dL.

e Without Hemolysis; plasma‐free Hb <50 mg/dL.

Plasma‐free hemoglobin measurement may not be widely available in every laboratory and requires turn over time. In addition, hemolysis can be rapidly progressive and its consequence would be catastrophic if not detected rapidly. CO‐Hb level by point‐of‐care co‐oximetry measurement would be particularly useful for early detection of hemolysis in this setting.

To our knowledge, the relationship of pfHb and CO‐Hb in patients receiving ECMO who develop mild‐to‐moderate degree of hemolysis has never been reported. Our finding provides a continuum of pfHb value against the CO‐Hb level in a cohort of patients receiving ECMO. While hypothesis generating, our finding is limited by a small number of patients who had hemolysis while receiving ECMO. However, it is of note that only 5.8% and 5.6% of patients receiving VV ECMO and VA ECMO, respectively, reported to ELSO‐developed hemolysis. A larger scale prospective evaluation would be warranted.

In summary, we present two patients with different degree of ECMO‐related hemolysis and endogenous carbon monoxide production. A positive correlation between CO‐Hb and pfHb may exist in ECMO patients with hemolysis. From our results, in patients receiving ECMO, CO‐Hb values of 3% appear to be discriminative for significant hemolysis.

## Authorship

Both authors contribute to the manuscript equally.

## Conflict of Interest

None declared.
